# Sensing Performance of Precisely Ordered TiO_2_ Nanowire Gas Sensors Fabricated by Electron-Beam Lithography

**DOI:** 10.3390/s130100865

**Published:** 2013-01-11

**Authors:** Wei-Cheng Tian, Yu-Hsuan Ho, Chao-Hao Chen, Chun-Yen Kuo

**Affiliations:** 1 Department of Electrical Engineering, National Taiwan University, Taipei 10617, Taiwan; 2 Graduate Institute of Electronics Engineering, National Taiwan University, Taipei 10617, Taiwan; E-Mails: d00943024@ntu.edu.tw (Y.-H.H.); r98945047@ntu.edu.tw (C.-H.C.); 3 Graduate Institute of Biomedical Electronics and Bioinformatics, National Taiwan University, Taipei 10617, Taiwan; E-Mail: d99945009@ntu.edu.tw

**Keywords:** temperature effect, TiO_2_ nanowire, gas sensors, electro-beam lithography

## Abstract

In this study, electron beam lithography, rather than the most popular method, chemical synthesis, is used to construct periodical TiO_2_ nanowires for a gas sensor with both robust and rapid performance. The effects of temperature on the sensing response and reaction time are analyzed at various operation temperatures ranging from 200 to 350 °C. At the optimized temperature of 300 °C, the proposed sensor repeatedly obtained a rise/recovery time (ΔR: 0.9 R_0_ to 0.1 R_0_) of 3.2/17.5 s and a corresponding sensor response (ΔR/R_0_) of 21.7% at an ethanol injection mass quantity of 0.2 μg.

## 1. Introduction

Gas sensors have been a focus of research in recent years for various applications, such as breath tests, environmental monitoring, indoor air quality, workplace health and safety, and homeland security. There have been numerous attempts to develop sensing devices with high sensitivity, stability, and rapid response [1–3]. In the past decade, conductometric-semiconducting metal-oxide gas sensors have attracted substantial interest because of their low cost and production flexibility, simplicity of use, multiplicity of detectable gases, and potential integration with complementary metal-oxide semiconductors (CMOS) or microelectromechanical system (MEMS) processes [4,5]. To increase the surface-to-volume ratio and enhance sensitivity, enabling the semiconducting conductance to be easily modulated by the target gas, fabrication processes and gas-sensing properties for metal-oxide nanostructures such as nanowires [6–13] and nanobelts [14–16] have been widely proposed in numerous studies. The electrical characteristics of nanostructures with high surface-to-volume ratios can be easily modified using structure shape and geometry, which likely allows various degrees of depletion from the charge carriers when gases are exposed to [17]. However, most current metal-oxide sensing films with nanostructures are fabricated by chemical synthesis and formed in irregular shapes. The sensing capability and repeatability of these irregular metal-oxide nanostructures is difficult to control and can cause unexpected measurement errors. This study uses a p-type TiO_2_ material to develop a unique metal-oxide-based gas sensor with engineered nanostructures using semiconductor nanotechnologies. Because of the combination of electron beam lithography and TiO_2_ thin-film deposition, TiO_2_-sensing nanowires with precisely controlled dimensions and geometries were obtained. This novel design demonstrated a reliable operation, and a fast sensing response in gas sensors for gas chromatography applications. The gas sensors were operated at temperatures ranging between 200 and 350 °C. Higher sensor operation temperatures typically led to stronger and faster sensing responses. However, the turning point for the response time of the proposed sensor was observed when its operation temperature was greater than 300 °C. In the following sections, the design, fabrication, and characterization of the proposed sensors are addressed. The effects of temperature on the thermal energy and coverage of the absorbed oxygen ions on the TiO_2_ surface are analyzed.

## 2. Experimental Section

**Figure 1.**
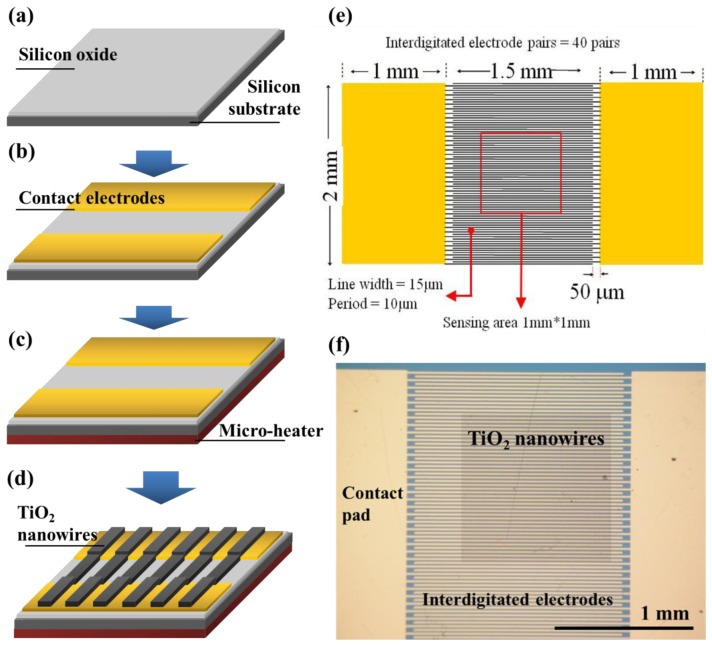
Device fabrication process: (**a**) Thermal oxidation of bulk Si wafer; (**b**) Deposition of Cr/Au for contact electrodes; (**c**) Deposition of Cr/Au on the backside of the Si subtract for the microheater; (**d**) E-beam lithography and TiO_2_ deposition followed by the lift-off process and (**e**) illustration of electrode and sensing film design on Si chips and (**f**) photo images of the TiO_2_ nanowire array bridging at the integrated electrodes.

The fabrication process of the TiO_2_ nanowire-based gas sensor is shown in Figure 1. Initially, a 0.3 μm thermal oxide was grown from a (100) p-type Si wafer, as shown in Figure 1(a). Next, interdigitated Cr/Au electrodes (Cr/Au thickness: 3/300 nm, width: 15 μm, gap between electrode fingers: 10 μm) were fabricated using the photolithography process on an oxidized Si substrate, as shown in Figure 1(b). Thereafter, 3-nm-thick Cr and 47-nm-thick Au thin films were blank-deposited on the back of the silicon substrate to create an integrated thin film microheater, as shown in Figure 1(c). The deposition rates for Cr and Au are 0.01 and 0.08 nm·s^−1^ respectively. Following the fabrication of the backside heater, standard e-beam lithography was used to pattern the top surface of the chip coated with a 0.6-μm-thick E-Beam resist Zep 520A (ZEON CSC Corp., Xinbi City, Taiwan) with the initial spin rate of 500 rpm for 5 s, followed by a speed ramping from 500 rpm to 5,000 rpm in 5 s, and a final spin rate of 3,000 rpm for 90 s. A 600 nm thick resist film was then formed. The lithography conditions (100 pA in current, 7 μsec/dot in dose time, and ZepN50 for developer) were optimized to produce nanowires with 100 nm and 300 nm in width and 1 μm in period in a 1 mm^2^ area. Next, the p-type TiO_2_ was deposited on the top of the chip with a sputter machine followed by the lift-off technique to form the TiO_2_ nanowire array (thickness: 300 nm, width: 100 nm, period: 1 μm), as shown in Figure 1(d). To enhance gas sensor sensitivity, the TiO_2_ nanowires were annealed at 450 °C for 1 h with a rapid thermal annealing machine prior to packaging. An optical image of the final device with the TiO_2_ nanowire array on its interdigitated electrodes is shown in Figure 1(f).

**Figure 2.**
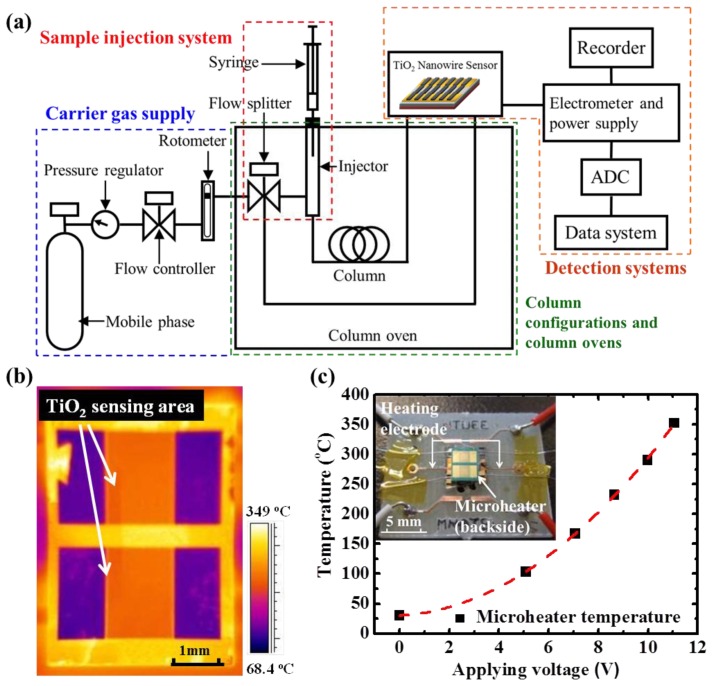
(**a**) Schematic of testing configuration; (**b**) thermal image of the sensor with an applied voltage of 9 V; (**c**) temperature responses of TiO_2_ nanowire gas sensor as a function of applied voltage to the backside of the microheater (inset of packaged sensor).

A testing platform, comprising the TiO_2_ gas sensor, a gas generation system, a commercial gas chromatography (GC) system (Agilent 5890, Santa Clara, CA, USA), a power supply, a high-precision ohmmeter, and an infrared (IR) detector (FLIR, Wilsonville, OR, USA), was constructed to evaluate the functionality of the proposed detectors. The testing setup is shown in Figure 2(a). Ambient air, filtered by the trapper, was pumped through an air compressor at a flow rate of 10 mL·min^−1^ and served as the carrier gas. The TiO_2_ gas detector was installed at the end of the separation column to provide a quantitative analysis. During the sensor characterization, the controlled amount of ethanol was injected multiple times with a fixed time interval in between injections to the commercial GC system and the individual ethanol peak was carried through the GC columns to our detector. The change of the sensing film resistance of our detector upon the exposure of the target gas was recorded. An IR detector was used to monitor the temperature distribution of the detector, to confirm uniform heating of the microheater during testing. Figure 2(b) shows the uniform temperature distribution inside the TiO_2_-sensing area with proper emissivity. In addition, temperature reading was verifiedwith an external thermocouple, which was attached underneath the microheater by a polyimide insulation layer.

**Figure 3.**
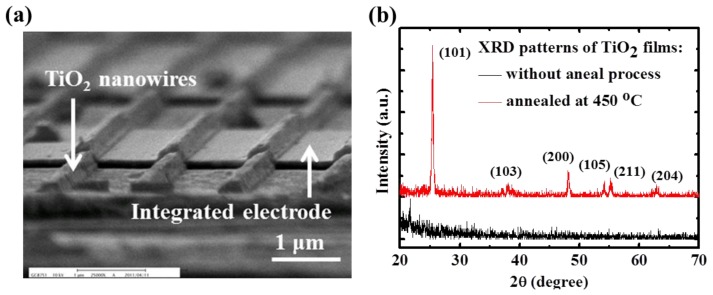
(**a**) SEM image of TiO_2_ nanowire gas sensor (**b**) XRD patterns of TiO_2_ thin film with 450 °C annealing for 1 h and without the annealing process.

The microheater was applied with various DC voltage values ranging from 0 to 11 V. The corresponding operation temperature (T, °C), which was a function of applied voltage (V, v), is shown in Figure 3(c), and it can be fitted well with a quadratic function, as T = 2.2 V + 2.42 V^2^. For gas-sensing applications, metal-oxide sensors must be measured at an operational temperature between 200 and 350 °C. To clarify the definition of the sensing response, the sensing response is defined as the normalized resistance change of TiO_2_ nanowires. The rising time was the time interval when there was an increase from 10% to 90%, and the recovery time was the interval for a reduction from 90% to 10% for sensing response.

## 3. Results and Discussion

A scanning electron microscope (SEM) image of the TiO_2_ nanowire array across the Au-interdigitated electrodes as the gas sensing film is shown in Figure 3(a). And the X-ray diffraction (XRD) patterns of the TiO_2_ films following annealing are shown in Figure 3(b). The as-grown film was identified as an amorphous type because no obvious peaks were located in the XRD plots. Following high-temperature annealing at 450 °C for 1 h, a clear peak at 2θ = 25.4 with a strong reflection from the (101) plane appeared, demonstrating the occurrence of polycrystallization. In addition to the main peak from the strong reflection of the (101) plane, several additional peaks, including reflections from the (103), (200), (105), (211), and (204) orientation planes, were observed. These observations confirmed that the annealed TiO_2_ was transformed from an amorphous phase to an anatase phase [18].

**Figure 4.**
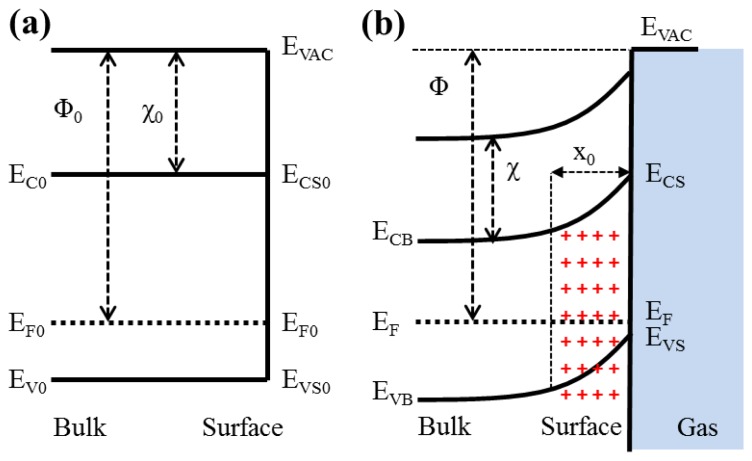
Band model showing the formation of a space charge region in a p-type TiO_2_ in presence of surface acceptor states before (**a**) and after (**b**) adsorption of ambient oxygen (E_vac_: potential of the electron in vacuum; x_0_: depletion width).

The proposed p-type TiO_2_ sensors are typically operated in temperatures ranging between 200 and 400 °C [4]. At these elevated temperatures, the chemisorption of atmospheric gases occurs at the surface and releases the holes to the valence band for association with the adsorption of ambient oxygen to create oxygen ions. As shown in Figure 4, this increase of holes in the valence band enhances the conductivity of p-type semiconducting TiO_2_ nanowires. After exposing the heated TiO_2_ nanowires to the reduction gases, the oxygen ions reacted with the reduction gases and resulted the reduction of holes in the valence band. Therefore, the resistance of the TiO_2_ nanowire array was increased [19].

To further elucidate the observations in the experimental data, the coverage of gas molecules on the TiO_2_ surface was examined. The coverage of gas molecules at equilibrium over a TiO_2_ surface can be expressed as [20]:

(1) $$\theta =\frac{k_{\text{ads}}}{k_{\text{des}}}\exp(\frac{\Delta H_{\text{chem}}}{kT})$$

where *k_ads_* and *k_des_* are the coefficient rates for adsorption and desorption, respectively; Δ*H_chem_* is the heat of the chemical adsorption; and *θ* is the surface coverage of the gas molecules. As shown in Equation (1), *θ* decreases with an increased temperature. Therefore, the coverage of oxygen ions, which is used to react with target gases, decreases with increased temperatures, and this declined coverage can reduce the sensitivity of the sensor.

**Figure 5.**
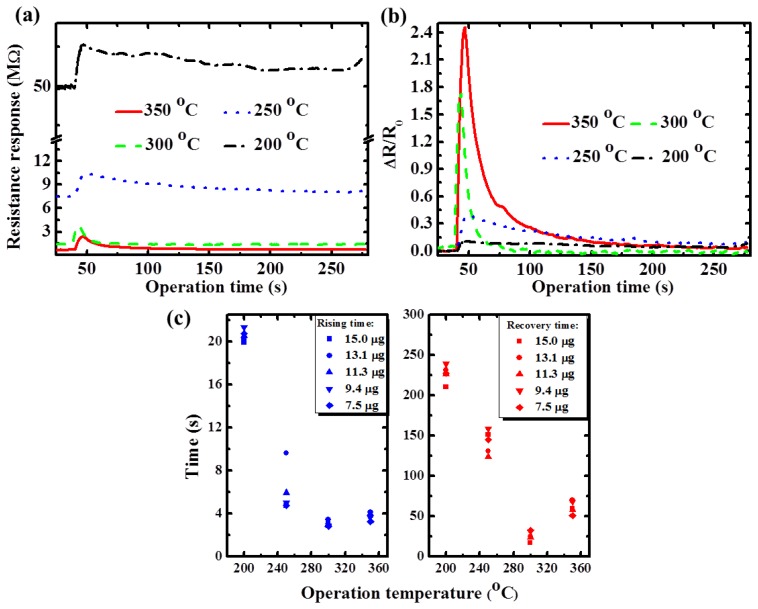
(**a**) Transient responses at various operation temperatures (with the injection absolute mass quantity of 7.5 μg); (**b**) normalized sensing responses at various operation temperatures of (a); and (**c**) reaction times of the TiO_2_ nanowire sensors tested at injection mass of 7.5 to 15.0 μg.

The detailed characterization results of the proposed p-type TiO_2_ nanowire sensor array are shown in Figure 5(a). When ethanol with the absolute mass quantity of 7.5 μg was injected onto the gas sensor through the GC system, the increase in nanowire resistance at low-operation temperatures was greater than that at high temperatures. This was caused by the rapid reduction of the initial resistance of the TiO_2_ nanowire array, which was due to the higher thermal energy of the TiO_2_ nanowire at higher temperatures. The normalized transient response (ΔR/R_0_) of the TiO_2_ nanowire sensor is shown in Figure 5(b). Based on the definition of sensing response (ΔR/R_0_), the device that operated at a high temperature had better performance than the device that operated at a low temperature. The relationship between operation temperature and response time is shown in Figure 5(c). Theoretically, the sensor responds faster when operated at higher temperatures because of its higher thermal energy. However, the lower surface coverage of the oxygen ions at higher temperatures, according to Equation (1), reduces the density of the reaction sites (oxygen ions) for ethanol.

In addition, the decreased density of the reaction sites increases the sensor response time because there is less possibility of collisions occurring between oxygen ions and target gas molecules. When the operation temperature of the TiO_2_ nanowire sensor is increased, two competing factors between the thermal energy of the material and the coverage of reaction sites affected the sensor response time; therefore, the sensor sensitivity did not increase linearly with the increased temperature. Thus, the TiO_2_ nanowire sensor had the fastest response at an operation temperature of approximately 300 °C because of competition between the higher thermal energy and the lower coverage of oxygen ions of the proposed TiO_2_ nanowire, which had a rising time of 3.21 s and a recovery time of 17.49 s.

**Figure 6.**
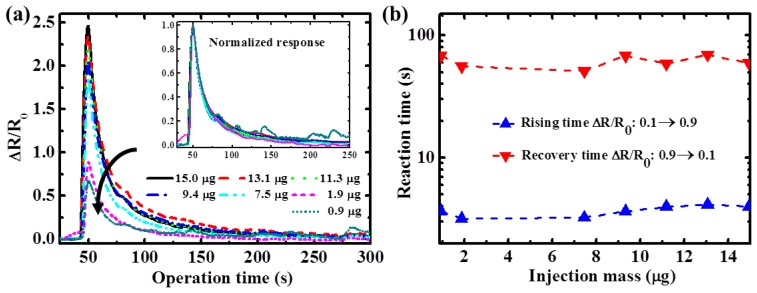
(**a**) Transient sensing responses and (**b**) reaction times of the TiO_2_ nanowire sensors at 300 °C with various ethanol injection mass quantities.

To characterize the influence of gas concentration for the sensor response, ethanol with various concentrations was injected into the TiO_2_ sensors at a constant temperature to evaluate the sensitivity and rising and recovery times. Figure 6(a) shows the transient response of the TiO_2_ nanowire sensor at 300 °C. A distinguishable resistance change ratio (ΔR/R_0_) was observed when the TiO_2_ sensors were exposed to ethanol. Sensing response of the TiO_2_ nanowire sensor to ethanol was higher at higher gas concentrations because there were more ethanol molecules that could react with the oxygen ions (the number of oxygen ions is assumed to be constant at the same temperature) over the TiO_2_ surface, compared to reactions with fewer ethanol molecules. The increased reactions between the ethanol and oxygen ions caused an increase in TiO_2_ resistance. However, the respective normalized signals of the sensing transients coincided with each other, as shown in the inset of Figure 6(a). This was because similar reaction rates between oxygen ions on a TiO_2_ surface and various concentrations of ethanol are obtained when the sensor is operated at a constant temperature. Therefore, the reaction time at a specific temperature was independent of ethanol concentration. As shown in Figure 6(b), both the rising and recovery times of the TiO_2_ nanowire sensors under various gas concentrations were nearly constant at 3.7 and 60 s, respectively.

**Figure 7.**
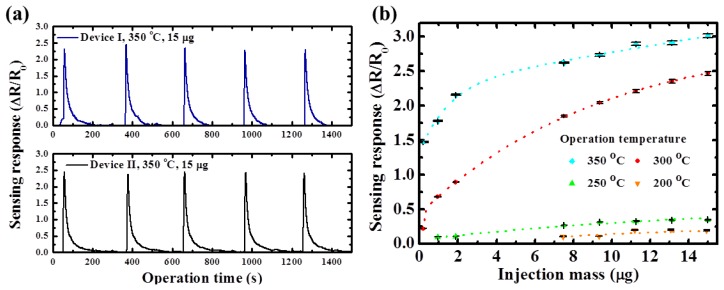
(**a**) Reliability test for devices (**b**) Effect of ethanol concentrations on the sensing response and corresponding standard deviations of TiO_2_ nanowire sensors under various operating temperatures.

Figure 7(a) shows the sensing responses for two different devices under the same operation temperature and the ethanol injection mass quantity. These two devices showed very similar sensitivity and close response time and the normalized sensing responses are approximately 1% different. The plot of the influence of various ethanol concentrations on the temperature-dependent gas sensing responses is shown in Figure 7(b). The corresponding standard deviation range is depicted as the error bar at each test condition. Since the finite oxygen ions were the reaction sites on the surface of the TiO_2_ nanowires, the resistance changes were gradually saturated when the ethanol concentration was sufficiently high. The proposed TiO_2_ nanowire sensors achieved superior repeatable sensing performances for ethanol. The normalized standard deviation values of the sensing responses for the various operation temperatures were 0.70% (350 °C), 0.82% (300 °C), 1.02% (250 °C), and 3.51% (200 °C), respectively. The sensing response (ΔR/R_0_) was equal to 21.7% at an ethanol injection mass quantity of 0.2 μg.

## 4. Conclusions

In conclusion, this study proposed a precisely aligned TiO_2_ nanowire-based gas sensor fabricated using e-beam lithography. The characteristics of TiO_2_ nanowire sensors under various operation temperatures were examined. Sensor response time was independent of ethanol concentration, and the optimized operating temperature was 300 °C with the proposed TiO_2_ nanowire design. With this optimized temperature, the rise and recovery times were reduced to 3.2 and 17.5 s, respectively, and the corresponding sensing response (ΔR/R_0_) was approximately 21.7% for the lowest ethanol injection mass of 0.2 μg.
